# Electromyographic Comparison of Traditional Fitness Machines, Outdoor Fitness Equipment Without Load Selectors, and Outdoor Fitness Equipment with Load Selectors in a Seated Chest Press Exercise in Trained Young Men

**DOI:** 10.3390/s24237740

**Published:** 2024-12-03

**Authors:** Tomás Abelleira-Lamela, Pablo Jorge Marcos-Pardo, J. Arturo Abraldes, Noelia González-Gálvez, Alejandro Espeso-García, Francisco Esparza-Ros, Raquel Vaquero-Cristóbal

**Affiliations:** 1 Facultad del Deporte, UCAM Universidad Católica de Murcia, 30107 Murcia, Spain; tabelleira@ucam.edu (T.A.-L.); ngonzalez@ucam.edu (N.G.-G.); aespeso@ucam.edu (A.E.-G.); 2 SPORT Research Group (CTS-1024), CIBIS (Centro de Investigación para el Bienestar y la Inclusión Social), University of Almeria, 04120 Almeria, Spain; 3 Department of Education, Faculty of Education Sciences, University of Almeria, 04120 Almeria, Spain; 4 Research Group Movement Sciences and Sport (MS&SPORT), Department of Physical Activity and Sport Sciences, Faculty of Sport Sciences, University of Murcia, 30720 San Javier, Spain; raquel.vaquero@um.es; 5 Injury Prevention in Sport Research Group, International Chair of Kinanthropometry, UCAM Universidad Católica de Murcia, 30107 Murcia, Spain; fesparza@ucam.edu

**Keywords:** outdoor gym, pectoralis major, resistance training, selectorized stack machine, surface EMG, upper limb muscle

## Abstract

Outdoor fitness equipment (OFE) are strength training installations comparable to those found in indoor gyms but are located outdoors with greater accessibility. However, the scientific evidence supporting their effectiveness remains limited. The objective of this study was to analyze and compare the electromyographic (EMG) activity of upper limb muscle groups during the use of a traditional seated chest press (SCP) machine, a classic OFE SCP (OFE-SCP), and a new OFE-SCP featuring a load selector system (BIOFIT-SCP). The sample was composed of 34 active young men. EMG activity of five muscle groups was analyzed: the anterior deltoid (AD), the clavicular (CP) and sternal (SP) heads of the pectoralis major, and the lateral (LHTB) and long heads of the triceps brachii (LongHTB), under different intensities. The OFE-SCP showed significantly lower EMG activity compared with the SCP and BIOFIT-SCP in all muscles and phases (*p* < 0.001). Significantly lower EMG activity for the SP in all three phases was found on the BIOFIT-SCP compared with the SCP (*p* < 0.001), but it was significantly higher for the LHTB and AD in the BIOFIT-SCP compared with the SCP during the full and concentric phases (*p* < 0.05 to *p* < 0.001). In conclusion, training with the OFE-SCP generates less EMG activity than traditional machine training, while, in general, BIOFIT-SCP proved effective for strength training comparable to the SCP.

## 1. Introduction

Strength training is becoming increasingly popular in the population thanks to the health benefits it brings to its practitioners [1,2]. Muscle mass gains and increased strength levels are the main objectives of most users who practice it [1,3]. Multiple training methods and tools can be used to train for strength, with guided machines being one of the most widely used [4].

However, in recent years, outdoor fitness equipment (OFE) has become popular as a more accessible alternative to guided machines in a fitness room [5,6]. These are known as sports equipment that are operated in a manner similar to traditional gym machines, although they are commonly located in public outdoor spaces such as parks [6,7,8]. Despite the popularity of OFE and the economic investment made for their installation and maintenance, there is some controversy regarding their effectiveness. In this line, while some studies indicate that training with OFE could improve body composition and strength production capacity in older adults [9], as well as improve physical functionality in seniors [10]; other studies indicate that training with this equipment may not be effective in adults and older adults, as a result of the reduced training intensity involved [11,12]. This is because traditional OFE are limited to working with one’s own body weight [12], so they do not allow for a controlled intensity progression within training sessions. Therefore, new models are emerging that allow the user to regulate the external load through a system that can be used to increase the weight, similar to that of traditional fitness room guided machines [9,13].

In recent years, machines that allow for pushing-based exercises in a guided manner have increased in popularity and can be found in both traditional guided machine training facilities [14] and OFE [5]. This is because these types of machines have been seen as an alternative to the use of free weights and can provide the same results but with greater safety and less reliance on exercise technique control [14]. Their use is common for both aesthetic purposes and to strengthen the upper body musculature, which is essential for performing daily tasks such as lifting or pushing objects [15].

Along this line, several previous studies have analyzed the efficacy of the seated chest press (SCP) machine by performing a surface electromyographic analysis (EMG) of the activation of the upper limb musculature during its execution [4,16,17,18]. The choice of the SCP machine was made because it is one of the most popular guided machines for push-work in fitness [19]. These studies have shown that the SCP is an effective machine for activating the main muscle groups involved in these pushing exercises, such as the anterior deltoid, the pectoralis major, and the triceps brachii [16,17,20].

In turn, within most OFE machine lines, SCP machines (OFE-SCP) are also included. However, so far, no studies have been found that analyze the effectiveness of OFE-SCP machines in terms of muscle activity of the a priori involved musculature. Therefore, for practical purposes, it is not known whether this machine could be effective in achieving adaptations in upper limb muscles after training with it. There is, however, a previous study that compared the EMG between traditional leg machines and the OFE leg machine, finding that, in general terms, the OFE showed less activation of the muscles involved in the lower limbs, which could be because it only allows working with one’s own weight [13].

Likewise, it has not been empirically proven whether the inclusion of an adjustable external weight in the OFE can improve the effectiveness of SCP machines. Only one previous study compared the EMG readings between a traditional guided leg machine and an OFE that allowed external weight adjustment, finding that at a similar intensity as a percentage of 1RM, a similar activation of the main lower limb musculature occurred, although the activation of the synergist musculature varied slightly because of the biomechanical differences between the exercises performed [13].

Therefore, the main objective of the present investigation was (a) to analyze and compare the EMG recorded in the upper limb muscle groups during the use of a SCP, OFE-SCP, and a push machine belonging to a new line of OFE that allows the external load to be regulated (BIOFIT-SCP); and (b) to determine the differences in the EMG of the upper limb muscle groups at 60 and 75% of 1RM in the SCP and BIOFIT-SCP machines. In relation to the hypothesis, it was proposed that: (a) the EMG of the upper limb musculature will be similar between the machines that allow the external load selection (SCP and BIOFIT-SCP), and activation will be greater in SCP and BIOFIT-SCP machines compared with a machine that only allows working with one’s own body weight (OFE-SCP); and (b) the EMG recorded will be greater at 75% of 1RM than in 60% of 1RM in SCP and BIOFIT-SCP machines.

## 2. Materials and Methods

### 2.1. Design

**Figure 1:**
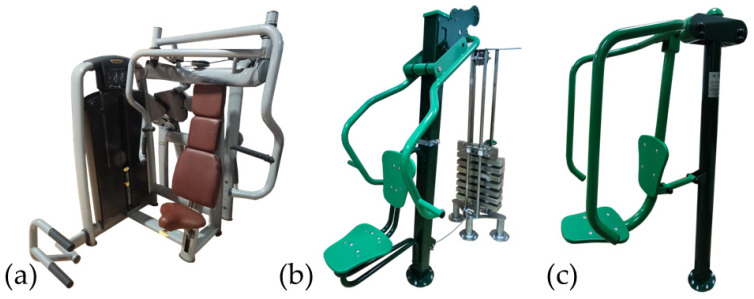
Fitness equipment used. (**a**) Seated chest press (SCP) (Technogym; Cesena, Italia); (**b**) outdoor fitness equipment seated chest press with selectorized system (OFE-SCP) (Entorno Urbano, Murcia, Spain); (**c**) outdoor fitness equipment seated chest press (BIOFIT-SCP) (Entorno Urbano, Murcia, Spain).

This research consisted of a randomized cross-sectional study. The 1RM was calculated on the SCP (Figure 1a) and the new BIOFIT seated chest press (BIOFIT-SCP), which is an OFE that has an external weight selector (Figure 1b). The calculation of the 1RM from each of the machines was done in a different session (session one and session two). Furthermore, the EMG activity of five muscle groups (anterior deltoid (AD), clavicular (CP) and sternal (SP) heads of the pectoralis major, and the lateral (LHTB) and long head of the triceps brachii (LongHTB)) was analyzed on three different types of chest press machines, i.e., SCP, OFE-SCP, and BIOFIT-SCP in another session (session three). In the SCP and BIOFIT-SCP machines, an EMG activity analysis was performed at 60 and 75% of the 1RM, while in the OFE-SCP machine, the analysis was performed with the subject’s own body weight. A period of 72 h was given between each of the evaluation sessions of each participant. The study utilized a specific research model, as illustrated in Table S1.

The machines selected for this research were: a machine commonly used in the fitness industry, the SCP model (Technogym; Cesena, Italy), with external load adjustment via a selectorized system [3] (Figure 1a); a machine belonging to a new line of OFE called BIOFIT-SCP (Entorno Urbano, Murcia, Spain), which allows the external load adjustment by means of a selectorized system [3], and which is patented (Patent registration for the OFE-SCP at the Spanish Patent and Trademark Office, application number: ES1296848Y; and patent registration for the external load selector, Spanish Patent and Trademark Office application number: P202231017) (Figure 1b); and a classic OFE machine line, corresponding with the same machine model, the Gemini (OFE-SCP) (Entorno Urbano, Murcia, Spain), which uses the user’s body weight as the external weight [10] (Figure 1c).

This research was conducted in accordance with the principles outlined in the Declaration of Helsinki and received approval from the institutional ethics committee (Ethical Approval Reference: CE111908). Before starting the study, all volunteers were informed about the different types of tests and voluntarily signed an informed consent form to participate in the present investigation.

### 2.2. Participants

A methodology utilizing the standard deviation (SD) was employed to determine the sample size [21]. For this analysis, RStudio version 3.15.0 was utilized. The calculations for sample size and statistical power were conducted based on the standard deviation of the variable peak pectoralis major EMG activity during the use of a SCP selectorized plate machine (SD = 63.82 µV) demonstrated by a sample of physically active young adults (n = 15) [18]. An estimated error (d) of 32.30 µV, along with a 95% confidence interval (95% CI), a statistical power of 95%, and a significance level of 0.05, indicated that a minimum of 34 participants was required for the study.

The study included a total of 34 male participants (mean age = 21.54 ± 1.80 years old; mean stretch stature = 176.62 ± 6.67 cm; mean body mass = 74.27 ± 9.99 kg). The inclusion criteria were: (1) being male, (2) a minimum of 2 years of experience using gym machines for training, along with currently engaging in strength training with these machines at least three times per week, and (3) being within the age range of 19 to 24 years old. The exclusion criteria were: (1) engaging in physical activity within 48 h before the initial session or between assessment sessions [19], (2) any recent injuries to the upper limbs or trunk within the previous 6 months [13], and (3) present a diagnosed muscle abnormalities or joint conditions [13].

### 2.3. Measurements

#### 2.3.1. Questionnaire

All the participants were asked to complete an ad hoc questionnaire on their socio-demographic characteristics, their experience in strength training, the use of guided machines, and possible injuries and pathologies suffered. This questionnaire was created based on previous studies [13].

#### 2.3.2. Anthropometric Measurements

For the measurement of the anthropometric variables stretch stature and body mass, the indications from the International Society for the Advancement of Kinanthropometry (ISAK) [22] were followed by an accredited ISAK level-2 anthropometrist. An HR001 portable stadiometer (Tanita, Arlington Heights, IL, USA) and a Tanita BC-545N scale (Tanita, Arlington Heights, IL, USA) were used. The variables were measured twice or three times if the difference between the first two was greater than 1%, with the final value being the mean or median, respectively.

#### 2.3.3. Assessment of 1RM

The 1RM was calculated in the SCP and BIOFIT-SCP machines. The OFE-SCP was discarded for this test because it does not have any external weight control system [12]. The order of use of the SCP and BIOFIT-SCP machines to determine the 1RM in each machine was randomized. The protocol recommended by the National Strength and Conditioning Association [23] was utilized for the calculation of 1RM in both machines. The participants performed a warm-up, comprised of a set with an intensity that allowed them to complete between 5 and 10 repetitions. Following a 1 min rest period, the intensity was increased by 10 to 20%, at which point they were required to perform at least three but no more than five repetitions. Following a 2 min rest period, the intensity was increased again by 10 to 20%. At this new intensity, participants were required to complete between two and three repetitions. After this, the intensity was adjusted upward by 10 to 20% for a single repetition attempt. Depending on whether the lift was successfully completed, the load was either increased or decreased by 5 to 10%, repeating this process until the 1RM was identified. A maximum of five attempts at varying intensities were performed, with four minutes between each attempt [23]. A total of 72 h later, the protocol was repeated to determine the 1RM on the other machine, following the prior randomization.

#### 2.3.4. Electromyographic (EMG) Analysis

Seventy-two hours after the second 1RM calculation session, the EMG signal was recorded in AD, CP, SP, LHTB, and LongHTB. The subjects performed, in a randomized order, five repetitions in the SCP machine with intensities of 60 and 75% of 1RM, five repetitions in the BIOFIT-SCP machine with intensities of 60 and 75% of 1RM, and five repetitions in the OFE-SCP machine with their own weight. This protocol followed the procedure of previous studies [13,24]. These intensities were selected for the SCP and BIOFIT-SCP machines as they are commonly used in different guided machines training methods [13,17].

**Figure 2:**
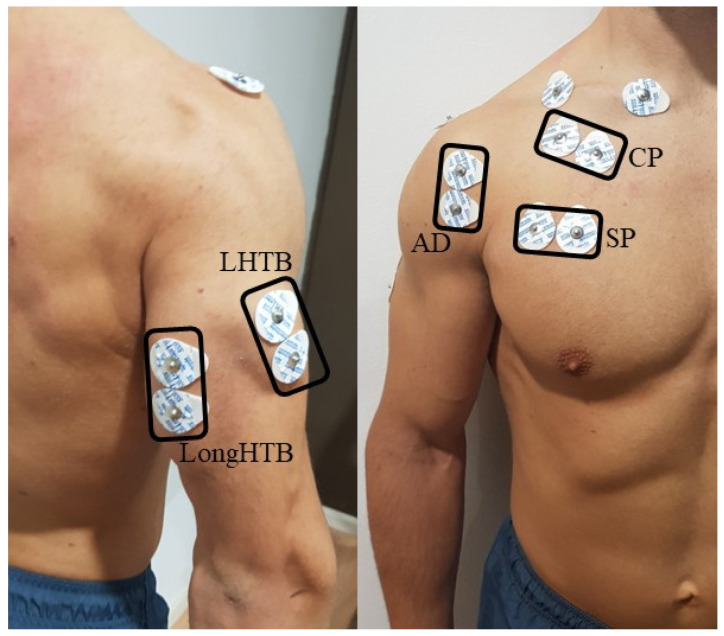
Electrode location on the right arm. AD = anterior deltoid; CP = clavicular pectoralis; LHTB = lateral head of the triceps brachii; LongHTB = long head of the triceps brachii; SP = sternal pectoralis.

The electrodes were arranged according to the recommendations from the Surface Electromyography for the Non-invasive Assessment of Muscles (SENIAM) project [25]. The areas where they were placed were shaved and cleaned with 96% alcohol and sterile gauze to avoid possible erroneous signals and reduced skin impedance [26]. The gel electrodes used were placed at a 2 cm center-to-center distance in the longitudinal direction of the muscle fibers [25]. Following the methodology from previous studies, bilateral symmetry was assumed during exercise execution, so all electrodes were located on the right side of the body in a standardized manner [27]. The parts recorded were CP, SP, LHTB, LongHTB, and AD (Figure 2). EMG activity was recorded during exercise using the MuscleLab surface electromyography system (Ergotest Innovation AS, Stathelle, Norway) at a sampling rate of 1500 Hz.

### 2.4. Randomization and Blinding

The sequence of machine usage for the 1RM assessment, the order of machine use for EMG analysis, and the initial 1RM intensity for each machine during EMG analysis were all randomized following the methodology of previous studies [28]. The procedure was conducted by the principal investigator in the presence of external researchers, utilizing a computer-generated random number table. All measurements were carried out following the randomization process.

The researchers who performed the EMG measurements were not involved in the 1RM calculation tests. In the measurements of EMG, the two investigators involved in the data collection were also blinded transversely, where one controlled the exercise execution technique and provided the guidelines for the development of the protocol, while the other focused exclusively on verifying the accuracy of the EMG data recording. This procedure was carried out following previous research [13].

### 2.5. Procedure

**Figure 3:**
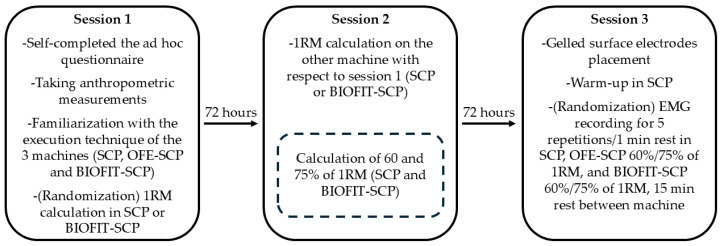
Flow diagram.

Figure 3 shows the procedures carried out during the three sessions in schematic form, following previous studies [13]. In session one, the volunteers filled out the ad hoc questionnaire, followed by the anthropometric measurements. Subsequently, the essential guidelines for proper exercise execution on each of the three machines (SCP, OFE-SCP, and BIOFIT-SCP) were provided to ensure they were familiar with the required execution technique for each exercise to facilitate accurate data collection. Specifically, they were informed that exercises on the various machines should be performed using a full range of motion., from 90° elbow flexion to full extension. On the SCP machine, the seat was placed at height number 3 so that the height of the seat to the grips was the same as on the BIOFIT-SCP machine.

Subsequently, participants carried out the 1RM calculation procedure using either the SCP or BIOFIT-SCP machine, with the order of execution assigned randomly. In the second session, the procedure was repeated to assess the 1RM in the other machine. Using the data collected from this procedure, the intensities corresponding to 60% and 75% of the 1RM were subsequently calculated for each participant on both the SCP and BIOFIT-SCP machines.

In the third session, following electrode placement, participants completed a warm-up that included 12 repetitions at 30% of 1RM, 10 repetitions at 50% of 1RM, 8 repetitions at 70% of 1RM, and 2 repetitions at 90% of 1 RM on the SCP, as this was the machine on which the participants had the greatest prior experience, similar to that reported in related studies [13,29]. EMG measurements were then initiated, with the assigned machine (SCP, OFE-SCP, or BIOFIT-SCP) selected randomly. For the SCP and BIOFIT-SCP machines, the intensity level (either 60% or 75% of 1RM) was also randomized. Participants were instructed to perform five repetitions on the initially assigned machine and intensity, maintaining a consistent speed throughout [26]. A 1 min rest was taken between each repetition. After a 15 min rest, the same protocol was repeated on the same machine but using the other intensity. Once the full protocol was completed on the first machine, participants rested for 15 min before beginning the protocol on the second machine [13,26]. In the case of OFE-SCP, a single set of five repetitions was carried out using the participant’s body weight, following the same procedure, since the external load could not be modified [6,10]. Rest periods were established based on previous methodologies to prevent fatigue from impacting the results [13,30].

For data analysis, all repetitions that had not been executed correctly from a technical point of view were discarded [13]. The correct execution was considered to involve the following assumptions: (a) that the repetition was performed with a constant rhythm throughout the entire range of motion, without stopping, (b) that a full range of motion from 90° elbow flexion to full extension was involved, and (c) that the buttocks were in constant contact with the seat and the feet were resting on the floor during the entire execution [31].

The data analysis was conducted using the methodology outlined in a previous study [26]. The first and last repetitions were excluded, and from the remaining three, the repetition with the highest root mean square (RMS) for the primary muscle group involved in the movement, the SP, was chosen, provided it was executed correctly. Data for all the muscles involved were recorded [13]. The data were processed using MuscleLab v10.5.67 software (MuscleLab Ergotest Innovation AS, Norway) by converting the raw amplified EMG signals into RMS values [32]. The RMS variable was measured during the entire repetition, as well as separately for its concentric and eccentric phases, to enable comparisons between machines, consistent with previous studies [33]. A MuscleLab twin-axis electrogoniometer (Ergotest Innovation AS, Stathelle, Norway) was utilized as a movement control variable to distinguish between the concentric and eccentric phases during exercise. The device was positioned on the participant’s left arm, near the elbow joint, on the outer side. The distal sensor was aligned with the forearm, while the fixed sensor was aligned with the upper arm [34]. Calibration was performed using a hand-held goniometer set at 90° of elbow flexion while the participant maintained a stable, relaxed position. The point of maximum elbow extension was verified in situ, allowing differentiation of the concentric phase as the data captured from the initial 90° flexion to full elbow extension and the eccentric phase as the data collected from maximum extension back to 90° flexion. The full phase encompassed the analysis of the entire range of motion, beginning and ending at 90° flexion [13].

All measurement sessions were spaced 72 h apart to prevent any potential fatigue effects [35]. All participants were scheduled to attend each session at 8:00 a.m. [13]. All measurements were conducted in the same room, under controlled conditions with a consistent temperature of 20 °C and a stable humidity level of 60%.

### 2.6. Statistical Analysis

Data analysis was conducted using SPSS software (Version 25; IBM Corporation, Armonk, NY, USA). The Kolmogorov–Smirnov test and Mauchly’s W-test were applied to assess data normality. Mean values and standard deviations were computed. To compare muscle activation across different machines and intensities, a repeated-measures ANOVA with Bonferroni correction was conducted. To control for Type I error, Bonferroni’s correction was applied, setting statistical significance at *p* = 0.005. A threshold of *p* < 0.05 was defined for acceptable error.

## 3. Results

Table 1 shows the 1RM strength test results for the traditional SCP and BIOFIT-SCP machines.

Table 2 shows the mean and standard deviation of the root mean square recorded in each of the muscles analyzed when using the SCP at 60 and 75% of 1RM, the BIOFIT-SCP at 60 and 75% of 1RM, and the OFE-SCP with their own weight. The muscle group that showed a greater RMS was the AD during the concentric phase and the LHTB during the eccentric phase, except for the OFE-SCP, in which the AD muscle group showed a higher activation in both phases. With respect to the complete phase, the highest activations were observed in both the AD and LHTB, with the most notable being those recorded in the BIOFIT-SCP. Regarding the pectoralis, it was observed that CP presented a greater activation than SP in the two types of OFE (BIOFIT-SCP and OFE-SCP), while in the SCP, the greatest activation was presented by SP. In the triceps, LHTB presented the highest activation in the three machines and during the three phases analyzed compared with the LongHTB.

**Figure 4:**
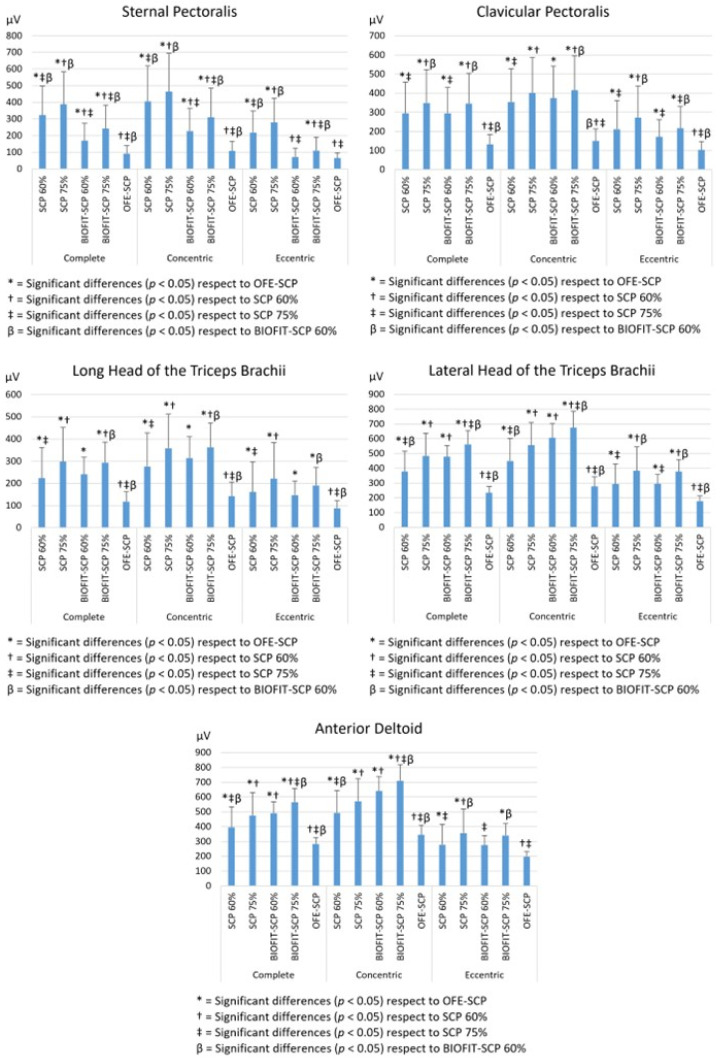
Differentiation of maximal electromyographic activity between different machines and 1RM intensities during the complete, concentric, and eccentric phases in different muscle groups.

Table 3, Table 4 and Table 5 and Figure 4 show the differences between machines and between different intensities in the full execution, concentric phase, and eccentric phase, respectively. At the same percentage of 1RM (60% 1RM vs. 60% 1RM, or 75% 1RM vs. 75% 1RM), the SCP presented a significantly greater activation of the SP in the three phases of execution (*p* < 0.001) and of the CP in the eccentric phase during execution at 75% of 1RM (*p* < 0.05) with respect to the RMS recorded in the BIOFIT-SCP. In the other phases and intensities, the CP did not present significant differences (*p* > 0.05). On the other hand, both LHTB (*p* < 0.01) and AD (*p* < 0.05) showed significantly higher activations in the full and concentric phases during the use of the BIOFIT-SCP with respect to the SCP at similar intensities, with no differences in the eccentric phase (*p* > 0.05). No difference in LHTB was found between SCP and BIOFIT-SCP at equal intensity (*p* > 0.05).

When comparing OFE-SCP with SCP and OFE-SCP with BIOFIT-SCP, a significantly lower activation was found in OFE-SCP for all muscle groups in the full execution (*p* < 0.001), concentric (*p* < 0.001) and eccentric phases (*p* < 0.05), except for the SP and AD during the use of BIOFIT-SCP at 60% of 1RM (*p* > 0.05) during the eccentric phase. When comparing each machine (SCP and BIOFIT-SCP) with each of the two different intensities, significant differences were found in all muscle groups and during all the phases (*p* < 0.01).

## 4. Discussion

The first objective of this research was to analyze and compare the EMG activity of the muscle groups of the upper limbs produced when exercising with the SCP, OFE-SCP, and BIOFIT-SCP machines. Based on previous studies, the performance of exercises involving internal rotation with shoulder abduction and flexion, together with elbow extension, regardless of the biomechanical differences between exercises, involve the same major muscle groups, for example, the pectoral musculature, the AD, or the triceps brachii [20,36]. Other studies have analyzed EMG activity in exercises such as the pec deck, chest press, or bench press, finding that they all activate the same major muscle groups, despite their technical differences, as they are all upper limb press exercises [19,20,36]. Furthermore, in all the exercises performed on stable guided machines, the stabilizing musculature of the trunk may be less involved, as they rely less on the postural control, compared with exercises with more degrees of freedom of movement, as proven in previous studies that showed significant differences in rectus abdominis activation, being lower during the use of a plate machine compared with a cable-based machine [20]. Based on this, in the present investigation, the decision was made to perform a comparison of the EMG activity with different types of machines, of the muscle groups directly involved in the movement, and not so much of the muscles that could be involved as stabilizers.

A notable finding of this research was that in all three machines, the muscle group that showed a greater RMS was the AD during the concentric phase and the LHTB during the eccentric phase in general. These results coincide with those found in similar works, with the AD being the upper limb muscle that showed the greatest activation during the use of the standing cable press [18]. As for the activation of the LHTB, it could be due to the need for stabilization of the movement, as proven in previous works in which stronger signals were recorded in exercises with a freer movement with respect to chest press machines for this muscle group [20].

An outstanding result of the present investigation was that when comparing EMG activity between machines with the same percentage of intensity, more specifically, SCP and the BIOFIT-SCP, the SP presented a significantly greater EMG signal in all phases recorded in the SCP with respect to the BIOFIT-SCP at the same relative intensities; while the CP showed greater activation in the SCP with respect to the BIOFIT-SCP, but only in the eccentric phase at 75% of the 1RM. This may be due to the fact that the bundles of the SP and CP parts have a recruiting advantage during horizontal glenohumeral flexion and may be due to the better alignment of the muscle fibers with respect to the direction of movement [19,33] during the execution of the SCP, but that the fibers did not maintain the same linearity during the execution of the BIOFIT-SCP. Furthermore, in previous studies where a comparison of the EMG activity of each muscle between exercises and grips was performed, it was observed that a bench press with a grip at 50% of the biacromial distance compared with a grip of 150% of the biacromial distance, presented a lower activation of the SP [4]. Thus, the fibers of the CP and SP were able to collaborate to a greater extent in all phases in the SCP machine with respect to the BIOFIT-SCP machine due to the closest grip in the exercise performed on traditional machines [33]. Given these preliminary results, questions remain as to the importance of fixed or adaptive grip distance during the use of this type of machinery on EMG activation in upper limb pushing exercises.

Continuing with the comparison between the SCP and the BIOFIT-SCP, in the case of the elbow extensors, the LongHTB showed no differences between machines at equal intensity, while the LHTB showed greater activation in the BIOFIT-SCP in the full and concentric phases, both at 60% and 75%, with no differences in the eccentric phase. Studies have justified the participation of the triceps as a synergist muscle in upper limb pushing exercises [20] and thrust exercises that increased its activation by elevating the firing frequency through the inclusion of a backrest that improved the intervention of the main musculature because it reduced the dependence of the stabilizing muscle during the exercise [18]. However, in most studies, the triceps brachii was analyzed uniquely as a muscle group, but locating the electrodes in only one of the muscle bellies [4,16,18,20], with this being the first article to analyze on different machines, the activation of two triceps muscle bellies, the LHTB and the LongHTB. The differences found between both machines in the EMG activity of the triceps brachii musculature may be conditioned by the angulation of the shoulder joint because the BIOFIT-SCP involves a shoulder flexion to a greater extent, while the SCP an adduction flexion, which would condition the EMG involvement of the triceps based on previous studies [19]. Given these promising results, future research is needed to analyze the biomechanics of both machines in order to confirm the results of the present investigation.

Similarly, when comparing EMG activation between SCP and BIOFIT-SCP, the AD showed greater activation in the BIOFIT-SCP in the full and concentric phases, both at 60% and 75%, with no differences in the eccentric phase. This could also be a consequence of the differences in shoulder abduction between the two machines. It has been shown that the AD is involved, to a greater extent, in exercises where the main movement consists of shoulder flexion, compared with those where the main movement is horizontal shoulder adduction [19]. In that sense, the movement in the BIOFIT-SCP is based on a shoulder flexion and an elbow extension with a small shoulder internal rotation, while the movement in the SCP involves a shoulder internal rotation and elbow extension, together with a steady shoulder abduction. Previous results on this issue are contradictory. Thus, while in some studies, it has been found that when comparing different types of plate-loaded chest press and cable-based strength training machines, the AD did not show significant differences between machines [18]. In other studies, it was found that the AD increased its activation during a chest press exercise as the inclination of the bench changed, leading to an increase in shoulder flexion [19]. The differences between studies may be due to the dependence of the execution technique in each of the variants of the exercises because the contribution of a specific muscle will depend on factors such as the specific movement performed in the joint and the anatomical position of the muscle [19]. These promising results indicate the need for future research to analyze the biomechanics of both machines to validate the findings of this study.

Another relevant result of this study was that, when comparing the EMG activity of the BIOFIT-SCP and SCP machines to the OFE-SCP, those machines equipped with an external weight selector showed significantly higher EMG values across all muscle groups, irrespective of the intensity and phase, with only a few exceptions. However, OFE has gained significant popularity in recent years [8,10,12]. No prior studies have been identified that have examined EMG activity using this type of machine, leaving its effectiveness uncertain [37]. However, prior research has indicated that the choice of intensity influences muscle fiber recruitment, thereby affecting the recorded signal [38]. Given that the traditional OFE line only permits exercises using one’s own body weight [12], the results of this study may be attributed to the fact that the participants’ body weight did not correspond to an intensity near 60% or 75% of their 1RM when using the OFE-SCP. Thus, based on the results of this study, OFE-SCP may have limited effectiveness in generating significant EMG activity [37]. Thus, based on the findings of this study, OFE-SCP may have limited effectiveness in generating significant EMG activity [39], being able to provide only an adequate stimulus in populations with low fitness levels, such as older, deconditioned individuals [40].

Based on the results found in the present investigation, the first hypothesis of this research can be partially accepted, as we found higher EMG activation in SCP and BIOFIT-SCP, as compared to OFE-SCP. However, the second part of the hypothesis, which hypothesized that the EMG activation between the machines with a load selector would be similar (SCP vs. BIOFIT-SCP), has to be rejected, as differences were found in four of the five muscle groups analyzed.

The second aim of this study was to assess the differences in EMG activity of upper limb muscle groups at 60% and 75% of the 1RM using machines that permit intensity adjustment (SCP and BIOFIT-SCP). The results indicated a rise in EMG activity across all the muscle groups analyzed when exercising at 75% of the 1RM compared to 60% of the 1RM for both the full range of motion as well as during the concentric and eccentric phases in the SCP and BIOFIT-SCP. The results are consistent with previous studies, which also reported an increase in EMG activity as intensity increased [41]. This occurs because, at higher intensities, there is an increase in both the recruitment of motor units and their firing frequency [38]. These results support the acceptance of the second hypothesis, as the EMG activity recorded at 75% of 1RM was higher than at 60% of 1RM in both SCP and BIOFIT-SCP conditions.

The primary practical implication of this research is the introduction of a new type of OFE, accessible to both trainers and users, supported by scientific evidence demonstrating its effectiveness in recruiting target muscles similarly to conventional gym equipment. Furthermore, the effectiveness of the OFE was enhanced by implementing an external load selection system. This approach not only ensured adherence to the principle of progressive intensity but also improved the control of training intensity when using these machines.

The main strength of the present research is that it is one of the first studies that has analyzed EMG activity in OFE with a load selector. As studies were not found that compared similar models of indoor and outdoor machines for upper limb exercises, the present study is a pioneer in this aspect. It is also one of the first articles that has analyzed the EMG activation generated by classic OFE, which works with the user’s own weight and whose shortcomings have been widely discussed. In addition, this work opens up new avenues of research by suggesting biomechanical and electromyographic analyses of the new patented systems and validities, offering opportunities to optimize their design and functionality in various contexts. In addition, it is one of the few works that includes both the eccentric and concentric phases, as well as the separate analysis of the sternal and clavicular parts of the pectoralis major muscle. In addition to the above, it is the only study that analyzed two of the triceps muscle bellies in guided machines.

Although this study offers a novel approach compared to prior research on the effectiveness of OFE, it is not free from limitations. Firstly, this study did not include an analysis of EMG results for antagonist muscles like the biceps brachii, latissimus dorsi, or other areas such as the lateral deltoid. The location of the electrodes in the back area makes measurement difficult because of the backrest of the machines. On the other hand, future work is encouraged to include an analysis of a larger number of muscle groups whenever possible. A second limitation was that the three machines presented slight variations in execution biomechanics due to the specific mechanics of each device, making direct comparisons between the exercises imprecise. However, it is important to note that studies with an electromyographic component often aim to highlight these biomechanical variations, providing valuable information on how different equipment or variations within the same exercise uniquely affect muscle activation. These comparisons are essential to understanding the nuances of muscle responses. The third limitation of this study was the exclusion of the maximum voluntary contraction test as a variable in the EMG analysis. This work compared the machines using the absolute values recorded. Including this variable could have provided insights into the percentage of activation of each muscle group relative to its maximum during each of the exercises assessed. In addition, it was not possible to calculate the 1RM in the OFE-SCP due to the impossibility of adjusting the load on the machine when working with one’s own body weight and mobilizing it with levers. As a final limitation, although the study incorporated an electrogoniometer for elbow joint monitoring during electromyographic signal recording, it did not incorporate a goniometric analysis of the elbow and shoulder joints, which might have provided information on joint angles and their relationship to muscle activation patterns. In addition, it could have provided findings by linking specific joint mechanics to differences in muscle recruitment across machines, such as the difference in angular velocity between shoulder and elbow and whether this changes the data recorded in the interfering muscle groups.

## 5. Conclusions

It was observed that the EMG activity presented by the current OFE designed to strengthen the upper limb musculature (OFE-SCP) is significantly lower than that of the SCP, a machine commonly used in strength training in fitness centers for the pectoralis, with higher activation in triceps. In contrast, classic OFE, which only allows self-loading work, was found to be less effective than seated chest press machines with external load selectors. Machines with external weight selectors (SCP and BIOFIT-SCP) consistently showed higher EMG activations across all muscle groups compared with self-loading OFE, suggesting that these may be less effective in generating sufficient muscle activation for hypertrophy and strength adaptations, except for populations with lower fitness levels. Thus, the study supports the idea that OFE with external load selectors represent a valuable tool for strength training, as they show EMG activity levels closer to those of conventional gym equipment. This innovation could improve the applicability of OFE in progressive training programs by allowing controlled intensity adjustments. Additionally, in general terms, a higher intensity implied a greater activation of the involved musculature. This study is a pioneer in the analysis of EMG activation in OFE with external load selection, as it included a separate analysis of the pectoralis major parts and two triceps muscle heads. Future studies should address the limitations of this research, proposing as future lines of research the inclusion of the analysis of antagonist muscles, the goniometric analysis of elbow and shoulder joint angles, as well as taking into account the differentiation of biomechanics of execution between machines, and the maximal voluntary contraction test, in order to know what percentage of activation is generated in each muscle group and to refine the understanding of muscle activation patterns. In addition, it will be necessary to expand the sample to be analyzed, taking into account the female population as well as the older population in future research in order to achieve a greater transfer to the population.

In summary, OFE equipped with external load selectors has the potential to bridge the gap between traditional self-weight OFE and gym-based strength training equipment, offering a controlled approach to muscle activation and adaptation for outdoor fitness applications.

## 6. Patents

This study presents data on two patents registered by the Spanish Patent and Trademark Office: a new machine designed for the outdoor fitness equipment line (code: ES2975897) and an innovative external load selection system of the exercise for this machine (code: ES2975886).

## Figures and Tables

**Table 1:** Results of the 1RM strength test.

	1RM	60% 1RM	75% 1RM
SCP (kg)	88.02 ± 20.72	57.21 ± 12.43	66.02 ± 15.54
BIOFIT-SCP (kg)	44.51 ± 15.06	26.71 ± 9.04	33.38 ± 11.30

1RM = one-repetition maximum; BIOFIT-SCP = BIOFIT seated chest press; SCP = traditional seated chest press.

**Table 2:** Root mean square recording of the electromyographic signal in the different machines with different 1RM intensities.

	SP (M ± SD)	CP (M ± SD)	LongHTB (M ± SD)	LHTB (M ± SD)	AD (M ± SD)
Complete					
SCP 60%	324.27 ± 173.47	294.47 ± 163.67	223.78 ± 138.17	377.78 ± 167.07	395.81 ± 181.21
SCP 75%	388.32 ± 195.96	348.72 ± 174.16	298.41 ± 153.99	483.80 ± 213.02	475.87 ± 216.16
BIOFIT-SCP 60%	170.39 ± 104.46	294.61 ± 135.61	240.59 ± 76.97	477.74 ± 209.51	491.45 ± 236.06
BIOFIT-SCP 75%	242.52 ± 140.62	345.74 ± 158.54	293.61 ± 92.29	562.39 ± 231.58	565.45 ± 256.92
OFE-SCP	92.13 ± 48.52	131.16 ± 52.86	118.06 ± 43.67	233.81 ± 97.10	282.89 ± 134.02
Concentric					
SCP 60%	405.24 ± 214.63	354.11 ± 174.17	276.03 ± 151.85	449.94 ± 203.93	492.81 ± 221.87
SCP 75%	464.00 ± 231.01	400.29 ± 185.58	357.95 ± 154.14	556.01 ± 241.40	570.43 ± 258.28
BIOFIT-SCP 60%	228.02 ± 137.01	373.41 ± 168.02	314.12 ± 96.52	606.76 ± 249.69	641.30 ± 290.93
BIOFIT-SCP 75%	310.05 ± 174.62	415.49 ± 179.38	362.90 ± 110.13	675.58 ± 269.82	709.09 ± 316.85
OFE-SCP	108.66 ± 58.08	150.04 ± 62.60	141.33 ± 65.01	277.29 ± 140.53	344.46 ± 156.46
Eccentric					
SCP 60%	218.49 ± 130.02	211.61 ± 149.48	160.86 ± 136.70	293.15 ± 152.20	277.87 ± 141.80
SCP 75%	279.58 ± 145.02	271.33 ± 166.85	220.94 ± 163.04	382.85 ± 183.09	355.95 ± 167.18
BIOFIT-SCP 60%	72.34 ± 53.03	171.94 ± 90.23	146.97 ± 63.24	294.86 ± 130.77	275.66 ± 173.07
BIOFIT-SCP 75%	110.50 ± 80.07	216.06 ± 115.29	190.81 ± 81.11	377.98 ± 148.77	340.04 ± 184.52
OFE-SCP	65.07 ± 30.76	103.21 ± 43.51	87.98 ± 35.07	177.75 ± 78.50	196.61 ± 113.77

AD = anterior deltoid; BIOFIT-SCP = BIOFIT seated chest press; CP = clavicular pectoralis; LHTB = lateral head of the triceps brachii; LongHTB = long head of the triceps brachii; OFE-SCP = classic OFE; SCP = seated chest press; SP = sternal pectoralis.

**Table 3:** Differentiation of peak electromyographic activity between different machines and intensities of 1RM during full execution.

Complete Repetition		SCP 60%	SCP 75%	BIOFIT-SCP 60%	BIOFIT-SCP 75%	OFE-SCP
Sternal pectoralis	SCP 60%	-	−64.05 ± 7.33 ^ⴕ^	153.88 ± 19.24 ^ⴕ^	81.75 ± 19.47 **	232.14 ± 25.54 ^ⴕ^
	SCP 75%		-	217.93 ± 21.28 ^ⴕ^	145.80 ± 19.93 ^ⴕ^	296.19 ± 29.60 ^ⴕ^
	BIOFIT-SCP 60%			-	−72.13 ± 8.12 ^ⴕ^	78.26 ± 15.25 ^ⴕ^
	BIOFIT-SCP 75%				-	150.39 ± 21.45 ^ⴕ^
	OFE-SCP					-
Clavicular pectoralis	SCP 60%	-	−54.25 ± 6.35 ^ⴕ^	−0.14 ± 15.64	−51.26 ± 16.01 *	163.31 ± 25.98 ^ⴕ^
	SCP 75%		-	54.11 ± 17.22 *	2.98 ± 16.46	217.55 ± 27.62 ^ⴕ^
	BIOFIT-SCP 60%			-	−51.12 ± 7.24 ^ⴕ^	214.57 ± 25.44 ^ⴕ^
	BIOFIT-SCP 75%				-	214.57 ± 25.44 ^ⴕ^
	OFE-SCP					-
LongHTB	SCP 60%	-	−74.63 ± 8.21 ^ⴕ^	−16.82 ± 21.09	−69.83 ± 22.03 *	105.71 ± 21.92 ^ⴕ^
	SCP 75%		-	57.81 ± 23.17	4.80 ± 23.21	180.34 ± 23.96 ^ⴕ^
	BIOFIT-SCP 60%			-	−53.01 ± 5.43 ^ⴕ^	122.53 ± 10.59 ^ⴕ^
	BIOFIT-SCP 75%				-	175.54 ± 13.57 ^ⴕ^
	OFE-SCP					-
LHTB	SCP 60%	-	−106.02 ± 12.90 ^ⴕ^	−99.96 ± 17.90 ^ⴕ^	−184.62 ± 22.30 ^ⴕ^	143.97 ± 17.26 ^ⴕ^
	SCP 75%		-	6.06 ± 17.69	−78.60 ± 17.92 **	249.99 ± 23.80 ^ⴕ^
	BIOFIT-SCP 60%			-	−84.66 ± 9.72 ^ⴕ^	243.92 ± 25.18 ^ⴕ^
	BIOFIT-SCP 75%				-	328.58 ± 28.63 ^ⴕ^
	OFE-SCP					-
Anterior deltoid	SCP 60%	-	−80.06 ± 13.14 ^ⴕ^	−95.64 ± 25.48 **	−169.64 ± 27.18 ^ⴕ^	112.92 ± 22.97 ^ⴕ^
	SCP 75%		-	−15.58 ± 25.56	−89.58 ± 25.83 *	192.98 ± 28.10 ^ⴕ^
	BIOFIT-SCP 60%			-	−74.01 ± 8.27 ^ⴕ^	208.56 ± 32.78 ^ⴕ^
	BIOFIT-SCP 75%				-	282.56 ± 36.24 ^ⴕ^
	OFE-SCP					-

BIOFIT-SCP = BIOFIT seated chest press; LHTB = lateral head of the triceps brachii; LongHTB = long head of the triceps brachii; OFE-SCP = classic OFE; SCP = seated chest press; * = *p* value < 0.05; ** = *p* value < 0.01; ⴕ = *p* value < 0.001.

**Table 4:** Differentiation of peak electromyographic activity between different machines and 1RM intensities during the concentric phase.

Concentric Phase		SCP 60%	SCP 75%	BIOFIT-SCP 60%	BIOFIT-SCP 75%	OFE-SCP
Sternal pectoralis	SCP 60%	-	−58.76 ± 8.28 ^ⴕ^	177.22 ± 23.83 ^ⴕ^	95.19 ± 24.31 **	296.58 ± 32.02 ^ⴕ^
	SCP 75%		-	235.98 ± 24.57 ^ⴕ^	153.95 ± 23.43 ^ⴕ^	355.34 ± 35.23 ^ⴕ^
	BIOFIT-SCP 60%			-	−82.03 ± 9.70 ^ⴕ^	119.36 ± 20.10 ^ⴕ^
	BIOFIT-SCP 75%				-	201.39 ± 26.60 ^ⴕ^
	OFE-SCP					-
Clavicular pectoralis	SCP 60%	-	−46.19 ± 8.71 ^ⴕ^	−19.30 ± 17.99	−61.38 ± 18.61 *	204.06 ± 28.57 ^ⴕ^
	SCP 75%		-	26.88 ± 20.15	−15.20 ± 18.40	250.25 ± 30.34 ^ⴕ^
	BIOFIT-SCP 60%			-	−42.08 ± 10.12 **	223.37 ± 28.25 ^ⴕ^
	BIOFIT-SCP 75%				-	265.45 ± 30.12 ^ⴕ^
	OFE-SCP					-
LongHTB	SCP 60%	-	−81.92 ± 10.20 ^ⴕ^	−38.09 ± 20.47	−86.88 ± 22.06 **	134.70 ± 22.52 ^ⴕ^
	SCP 75%		-	43.82 ± 20.25	−4.96 ± 20.38	216.61 ± 21.79 ^ⴕ^
	BIOFIT-SCP 60%			-	−48.78 ± 6.19 ^ⴕ^	172.79 ± 13.90 ^ⴕ^
	BIOFIT-SCP 75%				-	221.57 ± 16.35 ^ⴕ^
	OFE-SCP					-
LHTB	SCP 60%	-	−106.07 ± 15.19 ^ⴕ^	−156.82 ± 19.44 ^ⴕ^	−225.63 ± 23.34 ^ⴕ^	172.65 ± 19.97 ^ⴕ^
	SCP 75%		-	−50.75 ± 19.10	−119.56 ± 17.23 ^ⴕ^	278.72 ± 24.40 ^ⴕ^
	BIOFIT-SCP 60%			-	−68.82 ± 11.79 ^ⴕ^	329.47 ± 28.76 ^ⴕ^
	BIOFIT-SCP 75%				-	398.29 ± 31.03 ^ⴕ^
	OFE-SCP					-
Anterior deltoid	SCP 60%	-	−77.62 ± 15.64 ^ⴕ^	−148.49 ± 29.17 ^ⴕ^	−216.28 ± 31.89 ^ⴕ^	148.35 ± 29.06 ^ⴕ^
	SCP 75%		-	−70.87 ± 29.33	−138.66 ± 30.74 **	225.96 ± 32.72 ^ⴕ^
	BIOFIT-SCP 60%			-	−67.79 ± 11.52 ^ⴕ^	296.84 ± 39.90 ^ⴕ^
	BIOFIT-SCP 75%				-	364.62 ± 43.89 ^ⴕ^
	OFE-SCP					-

BIOFIT-SCP = BIOFIT seated chest press; LHTB = lateral head of the triceps brachii; LongHTB = long head of the triceps brachii; OFE-SCP = classic OFE; SCP = seated chest press; * = *p* value < 0.05; ** = *p* value < 0.01; ⴕ = *p* value < 0.001.

**Table 5:** Differentiation of peak electromyographic activity between different machines and 1RM intensities during the eccentric phase.

Eccentric Phase		SCP 60%	SCP 75%	BIOFIT-SCP 60%	BIOFIT-SCP 75%	OFE-SCP
Sternal pectoralis	SCP 60%	-	−61.09 ± 8.07 ^ⴕ^	146.15 ± 19.42 ^ⴕ^	107.99 ± 18.17 ^ⴕ^	153.41 ± 20.74 ^ⴕ^
	SCP 75%		-	207.24 ± 22.35 ^ⴕ^	169.08 ± 20.31 ^ⴕ^	214.50 ± 23.91 ^ⴕ^
	BIOFIT-SCP 60%			-	−38.16 ± 7.55 ^ⴕ^	7.26 ± 9.42
	BIOFIT-SCP 75%				-	45.43 ± 14.51 *
	OFE-SCP					-
Clavicular pectoralis	SCP 60%	-	−59.72 ± 5.72 ^ⴕ^	39.68 ± 16.51	−4.45 ± 15.46	108.40 ± 22.12 ^ⴕ^
	SCP 75%		-	99.39 ± 18.78 ^ⴕ^	55.27 ± 16.78 *	168.12 ± 25.04 ^ⴕ^
	BIOFIT-SCP 60%			-	−44.12 ± 8.30 ^ⴕ^	68.73 ± 11.99 ^ⴕ^
	BIOFIT-SCP 75%				-	112.85 ± 16.31 ^ⴕ^
	OFE-SCP					-
LongHTB	SCP 60%	-	−60.07 ± 6.92 ^ⴕ^	13.90 ± 22.49	−29.94 ± 23.21	72.89 ± 23.55 *
	SCP 75%		-	73.97 ± 26.27	30.13 ± 26.70	132.96 ± 28.10 ^ⴕ^
	BIOFIT-SCP 60%			-	−43.84 ± 6.50 ^ⴕ^	58.99 ± 10.72 ^ⴕ^
	BIOFIT-SCP 75%				-	102.83 ± 12.82 ^ⴕ^
	OFE-SCP					-
LHTB	SCP 60%	-	−89.70 ± 12.84 ^ⴕ^	−1.71 ± 18.26	−84.83 ± 20.58 **	115.40 ± 20.94 ^ⴕ^
	SCP 75%		-	87.99 ± 23.55 **	4.87 ± 24.39	205.11 ± 27.57 ^ⴕ^
	BIOFIT-SCP 60%			-	−83.12 ± 10.43 ^ⴕ^	117.11 ± 17.33 ^ⴕ^
	BIOFIT-SCP 75%				-	200.23 ± 19.84 ^ⴕ^
	OFE-SCP					-
Anterior deltoid	SCP 60%	-	−78.08 ± 14.19 ^ⴕ^	2.21 ± 24.45	−62.17 ± 23.57	81.26 ± 21.50 **
	SCP 75%		-	80.30 ± 25.32 *	15.91 ± 23.76	159.35 ± 23.67 ^ⴕ^
	BIOFIT-SCP 60%			-	−64.39 ± 8.76 ^ⴕ^	79.05 ± 27.79
	BIOFIT-SCP 75%				-	143.44 ± 30.00 ^ⴕ^
	OFE-SCP					-

BIOFIT-SCP = BIOFIT seated chest press; LHTB = lateral head of the triceps brachii; LongHTB = long head of the triceps brachii; OFE-SCP = classic OFE; SCP = seated chest press; * = *p* value < 0.05; ** = *p* value < 0.01; ⴕ = *p* value < 0.001.

## Data Availability

The data presented in this study are available on request from the corresponding author due to privacy.

## Supplementary Materials

The following supporting information can be downloaded at: https://www.mdpi.com/article/10.3390/s24237740/s1, Table S1. Research model.

## References

[1] Armstrong R., Baltzopoulos V., Langan-Evans C., Clark D., Jarvis J., Stewart C., O’Brien T. (2022). An Investigation of Movement Dynamics and Muscle Activity during Traditional and Accentuated-Eccentric Squatting. PLoS ONE.

[2] Martín-Fuentes I., Oliva-Lozano J.M., Muyor J.M. (2020). Evaluation of the Lower Limb Muscles’ Electromyographic Activity during the Leg Press Exercise and Its Variants: A Systematic Review. Int. J. Environ. Res. Public Health.

[3] Escamilla R.F., Fleisig G.S., Zheng N., Lander J.E., Barrentine S.W., Andrews J.R., Bergemann B.W., Moorman C.T. (2001). Effects of Technique Variations on Knee Biomechanics during the Squat and Leg Press. Med. Sci. Sports Exerc..

[4] Muyor J., Rodríguez-Ridao D., Oliva-Lozano J.M. (2023). Comparison of Muscle Activity between the Horizontal Bench Press and the Seated Chest Press Exercises Using Several Grips. J. Hum. Kinet..

[5] Chow H., Ho C.H. (2018). Does the Use of Outdoor Fitness Equipment by Older Adults Qualify as Moderate to Vigorous Physical Activity?. PLoS ONE.

[6] Jansson A.K., Lubans D.R., Smith J.J., Duncan M.J., Haslam R., Plotnikoff R.C. (2019). A Systematic Review of Outdoor Gym Use: Current Evidence and Future Directions. J. Sci. Med. Sport.

[7] Abelleira-Lamela T., Vaquero-Cristóbal R., González-Gálvez N., Esparza-Ros F., Espeso-García A., Marcos-Pardo P.J. (2021). Sagittal Spine Disposition and Pelvic Tilt during Outdoor Fitness Equipment Use and Their Associations with Kinanthropometry Proportions in Middle-Aged and Older Adults. PeerJ.

[8] Chow H., Wu D.-R. (2019). Outdoor Fitness Equipment Usage Behaviors in Natural Settings. Int. J. Environ. Res. Public Health.

[9] Marcos-Pardo P.J., Espeso-García A., Vaquero-Cristóbal R., Abelleira-Lamela T., González-Gálvez N. (2024). The Effect of Resistance Training with Outdoor Fitness Equipment on the Body Composition, Physical Fitness, and Physical Health of Middle-Aged and Older Adults: A Randomized Controlled Trial. Healthcare.

[10] Chow H., Chang K.T., Fang I.Y. (2021). Evaluation of the Effectiveness of Outdoor Fitness Equipment Intervention in Achieving Fitness Goals for Seniors. Int. J. Environ. Res. Public Health.

[11] Chow H.-W., Mowen A., Wu G. (2017). Who Is Using Outdoor Fitness Equipment and How? The Case of Xihu Park. Int. J. Environ. Res. Public Health.

[12] Liu Y.-C., Yang W.-W., Fang I.-Y., Pan H.L.-L., Chen W.-H., Liu C. (2020). Training Program with Outdoor Fitness Equipment in Parks Offers No Substantial Benefits for Functional Fitness in Active Seniors: A Randomized Controlled Trial. J. Aging Phys. Act..

[13] Abelleira-Lamela T., Marcos-Pardo P.J., Abraldes J.A., González-Gálvez N., Espeso-García A., Esparza-Ros F., Vaquero-Cristóbal R. (2024). Comparative Electromyographic Analysis in Leg Press of Traditional Fitness Equipment, Traditional Outdoor Fitness Equipment, and a New Model of Outdoor Fitness Equipment in Trained Young Men. Appl. Sci..

[14] Haugen M.E., Vårvik F.T., Larsen S., Haugen A.S., van den Tillaar R., Bjørnsen T. (2023). Effect of Free-Weight vs. Machine-Based Strength Training on Maximal Strength, Hypertrophy and Jump Performance—A Systematic Review and Meta-Analysis. BMC Sports Sci. Med. Rehabil..

[15] Hik F., Ackland D.C. (2019). The Moment Arms of the Muscles Spanning the Glenohumeral Joint: A Systematic Review. J. Anat..

[16] Coratella G., Tornatore G., Longo S., Esposito F., Cè E. (2019). Specific Prime Movers’ Excitation during Free-weight Bench Press Variations and Chest Press Machine in Competitive Bodybuilders. Eur. J. Sport Sci..

[17] López-Vivancos A., González-Gálvez N., Orquín-Castrillón F.J., Vale R.G.d.S., Marcos-Pardo P.J. (2023). Electromyographic Activity of the Pectoralis Major Muscle during Traditional Bench Press and Other Variants of Pectoral Exercises: A Systematic Review and Meta-Analysis. Appl. Sci..

[18] Signorile J.F., Rendos N.K., Heredia Vargas H.H., Alipio T.C., Regis R.C., Eltoukhy M.M., Nargund R.S., Romero M.A. (2017). Differences in Muscle Activation and Kinematics Between Cable-Based and Selectorized Weight Training. J. Strength Cond. Res..

[19] Trebs A.A., Brandenburg J.P., Pitney W.A. (2010). An Electromyography Enalysis of 3 Muscles Surrounding the Shoulder Joint during the Performance of a Chest Press Exercise at Several Angles. J. Strength Cond. Res..

[20] Cacchio A., Don R., Ranavolo A., Guerra E., McCaw S.T., Procaccianti R., Camerota F., Frascarelli M., Santilli V. (2008). Effects of 8-Week Strength Training with Two Models of Chest Press Machines on Muscular Activity Pattern and Strength. J. Electromyogr. Kinesiol..

[21] Bhalerao S., Kadam P. (2010). Sample Size Calculation. Int. J. Ayurveda Res..

[22] Esparza-Ros F., Vaquero-Cristóbal R., Marfell-Jones M.J. (2019). International Standards for Anthropometric Assessment.

[23] Miller T.A. (2012). NSCA’s Guide to Tests and Assessments.

[24] Duffey M.J., Challis J.H. (2007). Fatigue Effects on Bar Kinematic during the Bench Press. J. Strength Cond. Res..

[25] Stegeman D.F., Hermens H.J. (2007). Standards for Surface Electromyography: The European Project “Surface EMG for Non-Invasive Assessment of Muscles (SENIAM)”. Enschede Roessingh Res. Dev..

[26] Da Silva E.M., Brentano M.A., Cadore E.L., De Almeida A.P.V., Kruel L.F.M. (2008). Analysis of Muscle Activation During Different Leg Press Exercises at Submaximum Effort Levels. J. Strength Cond. Res..

[27] Snarr R.L., Esco M.R. (2013). Electromyographic Comparison of Traditional and Suspension Push-Ups. J. Hum. Kinet..

[28] Schoenfeld B.J., Contreras B., Willardson J.M., Fontana F., Tiryaki-Sonmez G. (2014). Muscle Activation during Low- versus High-Load Resistance Training in Well-Trained Men. Eur. J. Appl. Physiol..

[29] Andersen V., Fimland M.S., Mo D.A., Iversen V.M., Vederhus T., Rockland Hellebø L.R., Nordaune K.I., Saeterbakken A.H. (2018). Electromyographic Comparison of Barbell Deadlift, Hex Bar Deadlift, and Hip Thrust Exercises: A Cross-over Study. J. Strength Cond. Res..

[30] Gonzalez A.M., Ghigiarelli J.J., Sell K.M., Shone E.W., Kelly C.F., Mangine G.T. (2017). Muscle Activation during Resistance Exercise at 70% and 90% 1-Repetition Maximum in Resistance-Trained Men. Muscle Nerve.

[31] Mausehund L., Werkhausen A., Bartsch J., Krosshaug T. (2022). Understanding Bench Press Biomechanics—The Necessity of Measuring Lateral Barbell Forces. J. Strength Cond. Res..

[32] Van den Tillaar R., Andersen V., Saeterbakken A.H. (2019). Comparison of Muscle Activation and Kinematics during Free-Weight Back Squats with Different Loads. PLoS ONE.

[33] Albarello J.C.d.S., Cabral H.V., Leitão B.F.M., Halmenschlager G.H., Lulic-Kuryllo T., da Matta T.T. (2022). Non-Uniform Excitation of Pectoralis Major Induced by Changes in Bench Press Inclination Leads to Uneven Variations in the Cross-Sectional Area Measured by Panoramic Ultrasonography. J. Electromyogr. Kinesiol..

[34] Akima H., Maeda H., Koike T., Ishida K. (2021). Effect of Elbow Joint Angles on Electromyographic Activity versus Force Relationships of Synergistic Muscles of the Triceps Brachii. PLoS ONE.

[35] Picerno P., Iannetta D., Comotto S., Donati M., Pecoraro F., Zok M., Tollis G., Figura M., Varalda C., Di Muzio D. (2016). 1RM Prediction: A Novel Methodology Based on the Force–Velocity and Load–Velocity Relationships. Eur. J. Appl. Physiol..

[36] Stastny P., Gołaś A., Blazek D., Maszczyk A., Wilk M., Pietraszewski P., Petr M., Uhlir P., Zajac A. (2017). A Systematic Review of Surface Electromyography Analyses of the Bench Press Movement Task. PLoS ONE.

[37] Mehr K. (2013). Surface Electromyography in Orthodontics—A Literature Review. Med. Sci. Monit..

[38] Cormie P., McGuigan M.R., Newton R.U. (2011). Developing Maximal Neuromuscular Power Part 1-Biological Basis of Maximal Power Production. Sports Med..

[39] Schwanbeck S.R., Cornish S.M., Barss T., Chilibeck P.D. (2020). Effects of Training with Free Weights Versus Machines on Muscle Mass, Strength, Free Testosterone, and Free Cortisol Levels. J. Strength. Cond. Res..

[40] Kim D.-I., Lee D.H., Hong S., Jo S., Won Y., Jeon J.Y. (2018). Six Weeks of Combined Aerobic and Resistance Exercise Using Outdoor Exercise Machines Improves Fitness, Insulin Resistance, and Chemerin in the Korean Elderly: A Pilot Randomized Controlled Trial. Arch. Gerontol. Geriatr..

[41] Snyder B.J., Fry W.R. (2012). Effect of Verbal Instruction on Muscle Activity Duringthe Bench Press Exercise. J. Strength. Cond. Res..

