# Supplementary material for: Electromyographic Comparison of Traditional Fitness Machines, Outdoor Fitness Equipment Without Load Selectors, and Outdoor Fitness Equipment with Load Selectors in a Seated Chest Press Exercise in Trained Young Men
Source: Sensors (Basel). 2024 Dec 3;24(23):7740. doi: 10.3390/s24237740 (PMC11644873; doi:10.3390/s24237740)
Supplement: Supplementary file 1 [file sensors-24-07740-s001.zip › sensors-3339796-supplementary.pdf]

Table S1. Research model

Variable type	Construct	Variables
Independent	Load applied	60%1RM, 75%1RM
Independent	Equipment	Chest Press (SCP)<br>Geminis (OFE-SCP)<br>BIOFIT-Park Chest Press (BIOFIT-SCP)
Dependent	Electromyographic activation	RMS of Anterior deltoid<br>RMS of Clavicular pectoralis<br>RMS of Lateral head of the triceps brachii<br>RMS of Long head of the triceps brachii<br>RMS of Sternal pectoralis

1RM : One-repetition maximum; RMS : Root mean square
